# Supplementary material for: Packaging contests between viral RNA molecules and kinetic selectivity
Source: PLoS Comput Biol. 2022 Apr 1;18(4):e1009913. doi: 10.1371/journal.pcbi.1009913 (PMC9022832; doi:10.1371/journal.pcbi.1009913)
Supplement: S1 Text — (PDF) [file pcbi.1009913.s001.pdf]

I. SMOLUCHOWSKI THEORY OF BIMOLECULAR REACTIONS

The expression used for the on-rates $W_{n,n+1}$ used in the main text is based on Ch.8 of the lecture notes "Non-Equilibrium Statistical Mechanics", UIUC, by Klaus Schulten, which we briefly review here for the benefit of the reader.

According to Smoluchowski theory, the steady-state particle current J produced by a bi-molecular reaction that is governed by a radial interaction potential $U(r)$ is given by

$$J = 4\pi r^2 D \left(\partial_r + \beta \partial_r U(r)\right) p(r)$$

where r is the separation between two reacting particles 1 and 2, $p(r)$ the two-particle density distribution function, D the sum of the diffusion coefficients of the two particles and $\beta = 1/k_b T$. In the large r limit, $p(r)$ must approach the product $c_1 c_2$ of the concentrations of the reactants. Next, if the the reaction surface is a sphere of radius R_0 then the particle current must equal $J = 4\pi R_0^2 w p(R_0)$ with w a measure of the reaction rate. Inserting the expression for the current and using the fact that the current is constant produces a first-order differential equation for $p(r)$:]

$$4\pi r^2 D \left(\partial_r + \beta \partial_r U(r)\right) p(r) = 4\pi R_0^2 w p(R_0)$$

with solution

$$p(r) e^{\beta U(r)} = c_1 c_2 - \frac{R_0^2 w p(R_0)}{D} \int_r^\infty dr' \frac{e^{\beta U(r')}}{r'^2}$$

Evaluation at $r = R_0$ gives

$$p(R_0) = \frac{c_1 c_2 e^{-\beta U(R_0)}}{1 + (R_0^2/D) w e^{-\beta U(R_0)} \int_{R_0}^\infty dr' \frac{e^{\beta U(r')}}{r'^2}}$$

The corresponding reaction rate $4\pi R_0^2 w p(R_0)$ is

$$Rate = \frac{4\pi R_0^2 w c_1 c_2 e^{-\beta U(R_0)}}{1 + (R_0^2/D) w e^{-\beta U(R_0)} \int_{R_0}^\infty dr' \frac{e^{\beta U(r')}}{r'^2}}$$

Expressing the reaction rate as $k c_1 c_2$ with k the on-rate rate constant gives

$$k = \frac{4\pi}{\frac{e^{\beta U(R_0)}}{4\pi R_0^2 w} + (1/D) \int_{R_0}^\infty dr' \frac{e^{\beta U(r')}}{r'^2}}$$

For a diffusion-limited reaction, the first term in the dominator can be neglected with respect to the second term. If one also sets the radial potential $U(r)$ to zero then this produces the standard expression $k = 4\pi D R_0$ of Smoluchowski theory. For the present case, the potential $U(r)$ describes the binding of a capsomer to the aggregate. As a simple model, we set $U(r)$ equal to the energy cost $\Delta\Delta E$ of binding a pentamer for $r - R_0$ less than an interaction range δ and equal to zero for $r - R_0$ larger than δ. Carrying out the integral over a r' gives:

$$k \simeq \frac{4\pi}{e^{\beta \Delta\Delta E} \left(\frac{1}{4\pi R_0^2 w} + \frac{(\delta - R_0)}{D R_0^2} \right) + 1/(D R_0)}$$

For $\Delta\Delta E$ negative and large compared to the thermal energy, the first term in the denominator can be neglected and one recovers the usual Smoluchowski rate, denoted by λ in the main text. For $\Delta\Delta E$ positive and large compared to the thermal energy, the first term dominates and the reaction rate can be approximated as $k \simeq \frac{\lambda}{\frac{D/R_0}{4\pi w} + \frac{(\delta - R_0)}{R_0}} e^{-\beta \Delta\Delta E}$. For diffusion-limited reactions, the first term in the denominator is small compared to the second term. Assuming that $\frac{(\delta - R_0)}{R_0}$ is of the order on one produces the kinetic Monte-Carlo rates used in the text.
