# Supplementary material for: Packaging contests between viral RNA molecules and kinetic selectivity
Source: PLoS Comput Biol. 2022 Apr 1;18(4):e1009913. doi: 10.1371/journal.pcbi.1009913 (PMC9022832; doi:10.1371/journal.pcbi.1009913)
Supplement: Attachment — Submitted filename: Response_PLOS(2)_Jan.18.pdf [file pcbi.1009913.s002.pdf]

Referee 1

1. The authors explain the assembly of capsid proteins around ssRNA based on the heterogenous nucleation. However, they discuss that "the spatial distribution of packaging signal is likely to play an important role." If the process is based on the nucleation and growth, then why is the distribution of packaging signals important? After the capsid is nucleated in one of the packaging signals, does it still matter that there are other packaging signals if the process is based on the nucleation and growth? One might think about the process of multi-nucleation on different segments of the RNA, but it seems that the authors do not agree with the en mass assembly or multi nucleation growth. For the en mass mechanism and the multi-nucleation process see ACS Nano 14, 3170-3180 (2020).

Our original text was unclear. With the term "heterogeneous nucleation" we meant that assembly of virions in solution takes place on ssRNA molecules as opposed to the ``homogeneous nucleation" of empty capsids that can take place anywhere in solution. We removed the term. The referee asks, in this context, why individual packaging signals on viral RNA molecules would not be able to initiate assembly and points out - correctly - that if that was the case then the spatial distribution of packaging signals should not matter. We use the term "packaging signal" for RNA hairpins of less than 10 nucleotides with affinity for capsid proteins. Individual packaging signals of this type are *not* supposed to be able to trigger virion assembly. The reason is that any mRNA strand involves thousands of nucleotides and has numerous hairpins, which are often quite similar. Purely by random chance, a host mRNA molecule can have hairpins that are either equal to or closely similar to a viral packaging signal. A capsid assembly process that has to pick out a few genomic viral ssRNA molecules from among a large pool of host mRNA molecules necessarily has to involve multiple, cooperative packaging signals in proximity of each other. This is discussed in the revised manuscript in the introduction section.

The referee asks why we disagree with the *en-masse* and multi-nucleation scenarios for viral assembly. We do not and this is also due to lack of clarity. Our work was motivated by the asymmetric reconstruction of the MS2 virus by Dykeman et al. who found that a large section of the viral RNA genome rich in packaging signals reproducibly associated with roughly half of the interior surface of the capsid. They argued that the spatial distribution of packaging signals provides a "map" for the viral assembly process. Such a description only "works" if MS2 assembly is a protein-by-protein process rather than an *en-masse* or a multi-nucleation scenario. However, that need not be the case for other viruses. Assembly experiments on the bromovirus family appear to be consistent with an *en-masse* or a multinucleation scenario. The aim of the manuscript is to propose an assembly scenario for viruses, such as MS2, for which there is evidence that the spatial distribution of packaging signals guides the assembly process in a correlated fashion. We now also include a discussion of RNA selection by the TMV and HIV-1 viruses during nucleation. The new text clarifies this important point in the introduction section.

2. The authors ignore the importance of the length of genome, which is often longer than mRNA. Recently, it was shown that the capsids assembled around shorter RNAs are unstable, see Small 2020, 16, 2004475. See also Physics Reports 847, 1-102 (2020) and many references therein.

This is quite true. The proposed model focuses on how the secondary structure of packaging signals on the surface of the condensed genome guide viral assembly. It assumes that the RNA material that fills the interior such that the size of the condensed RNA molecule has the right size so it can be covered by the capsid. As the referee points out, if the RNA molecule is too short (or too long!) then that can destabilize assembly. Yes, in a packaging competition experiment the length of the competing RNA molecules definitely should play a role and, as mentioned, the model does not include that. We now clarify this in the introduction and the conclusion. There are two stages for RNA selection during assembly. The first nucleation stage involves the appearance in solution of critical nuclei composed of a small number of capsid proteins associated with an RNA molecule. A kinetic description is appropriate for this stage. The second elongation stage is the completion of the virion by addition of capsid proteins diffusing in from solution. If an RNA molecule has the wrong length so capsid formation cannot complete then virion particles will not be able to form. Equilibrium free energy minimization rather than kinetics is the natural language for describing RNA selection during this second stage. Yes, there is an extensive literature on the free energy of virions and we should definitely have included the very nice Physics Reports review article and the Small article as we now do. *However, the focus of the paper is on the first assembly stage and the kinetic RNA selection mechanism.* The new text now extensively discusses in the conclusion this two-stage aspect and how the model can be extended to include competition between RNA molecules with different lengths.

3. If there are a few long branches in an RNA, it is not obvious if MLD is still a good "predictor" of RNA branchiness. Have you seen the work of van der Schoot and collaborators? For example, they show how the free energy or osmotic pressure due to the presence of RNA changes with the degree of RNA branching, see for example Phys. Rev. E 94, 022408, (2016). Since the base-pairing increases the stiffness of RNA segments, sometimes the linear polymers win over the branched ones to attract proteins (J. Phys.: Condens. Matter 30, 044002 (2018)). Also see The Journal of Physical Chemistry B, 119, 13991-14002 (2015) how branchedness and length contribute to the competition experiments.

The original text did not make clear that the MLD in the article *only* refers to the section of the gRNA molecule that carries the packaging signals. This MLD must be distinguished from the "global" MLD of the molecule as a whole that has been discussed in a number of papers. The model focuses exclusively on the early stages of nucleation-and-growth when the packaging section of a gRNA molecule interacts with only a limited number of capsid proteins. The osmotic pressure and the global MLD are very important for completion of the capsid, not for this early nucleation stage. The work of van der Schoot as well as the other articles are based on minimization

of the free energy and do not address the nucleation stage when the RNA that will be packaged is being selected. In the rewritten manuscript we now try to clarify this crucial point.

4. Can the authors explain why they believe that the RNA selection mechanism is purely kinetic and disappears after the system has reached full thermodynamic equilibrium. Several papers show that the encapsidation of branched polymers is energetically more advantageous than a linear one, see Elife 2, e00632 (2013) in addition to the paper mentioned in item 3.

We now summarize in the introduction experimental evidence that the selective action of packaging signals takes place during the early nucleation stage of virion assembly for the case of TMV, HIV-1, and MS2. We also point out the contrary case of the Bromoviruses. In the conclusion we give arguments why the assumption of full thermodynamic equilibrium for virions in general cannot hold under practical conditions, and why selection during elongation should be discussed in terms of free energy minimization.

We want to emphasize though that we presented a simple statistical-mechanical model for the kinetic selection of gRNA molecules and we stated the severe limitations and restrictions of the model. The sections that were added should, we hope, clarify the reasoning behind the choices for the model. These choices certainly can and should be questioned but we hope that the model can be judged on its own merits.

5. The authors find that the en-masse scenario is not a viable route for selective virion assembly. Have they seen the experiments of Tresset and collaborators, which show that for CCMV, en mass mechanism is a viable one (Nat. Commun. 9, 3071 (2018)), also see the simulations corresponding to his experiments in ACS Nano 14, 3170-3180 (2020).

The *en-masse* scenario may very well be a viable description for CCMV assembly! It is known that CCMV capsid proteins can encapsidate *just about any* ssRNA molecule with a length comparable to the viral RNA molecules. In fact, CCMV capsids have been proposed as an RNA delivery vehicle in mRNA-based medicine. Amazingly, in a packaging competition experiment, CCMV capsid proteins were found to encapsidate ssRNA molecules of the (related) BMV virus in preference to its own viral RNA. CCMV capsid proteins can even encapsidate synthetic polyelectrolyte molecules. How is CCMV able to "get away" with so little selectivity? The CCMV genome includes the gene of an enzyme (replicase) that makes copies of the viral genome. After a cell is infected, transcription of viral RNA could result in a massive amount of viral genome molecules, replicase proteins and capsid proteins. Under those conditions, viral assembly would not need to be RNA selective at all since the minority of particles that contain host mRNA instead of viral RNA simply are overwhelmed by virions. As discussed above, the paper proposes an assembly mechanism that is relevant only for those cases where assembly is highly RNA selective. The work of Tresset et al. is now discussed in the conclusions.

6. The authors state, “Such “specific” interactions operate against a background of a generic

electrostatic affinity between the negatively charged RNA nucleotides and positively charged residues of the capsid proteins." Why does it work against? Doesn't specific interaction facilitate the packaging, thus helping the electrostatic interactions?

This was again due to poor writing on our side. Yes, non-specific electrostatic interactions and specific RNA-protein interactions *both* facilitate packaging. We rewrote the paragraph.

Referee 2:

Mizrahi et al. examine the selective packaging of RNAs into viral capsids. Their model assumes capsids are built from 12 pentameric subunits and the RNA interacts along edges of the pentamers. They then examine packaging competitions between different shaped RNAs which can be characterized by their maximum ladder distance.

The work in this manuscript is similar to that done by Dykeman and Twarock on the assembly competition between viral genomic RNAs and host cellular mRNAs that would occur in vivo, but contains a couple of key differences which make it new.
1) The authors model treats the interaction between RNA and capsid as occurring between pentameric edges. This type of model would be applicable to insect viruses in the Nodaviridae family which construct a dodecahedral cage of ds-RNA. An assembly model looking at this type of interaction (along with a combinatorics enumeration of the RNA configurations) has not been constructed yet.
2) The model attempts to account for some of the effects of the geometry of the RNA secondary structure and its effects on RNA assembly efficiency which is lacking in previous models (e.g. Dykeman et al.).

Some comments on the science.
MAJOR
1) Enumeration/Combinatorics of the spanning trees. There is limited discussion on the overall numbers of spanning trees and the computational method how they were counted. The manuscript also leaves out an important discussion on the number of ways to embed a spanning tree. For example. The linear spanning tree case corresponds to the previous treatment of RNA paths in Dykeman et al. For this one case, there are ~ 64 ways to embed the linear spanning tree to obtain a Hamiltonian path. Similarly, there will be an equivalent treatment for the other spanning tree shapes which will each have a potentially different number of ways to embed the spanning tree into the dodecahedron protein shell resulting in a Hamiltonian path.

Paths are fairly easy to construct on the dodecahedron, so I don't think it should be to hard to enumerate the embedding options (only 64 for the linear case with less options I would guess for more complicated trees). But this is dependent on the Authors spanning tree enumeration procedure which they should discuss.

The Authors use the minimum energy embedding, where the pentamers are brought onto the RNA in an order which minimizes the energy at each step. This seems reasonable, but could have some issues if there is more than one path which does this. This would give an entropic term that needs to be accounted for where spanning trees with more path options that have the same energetic pathway are favored over others with less options. For example, in Fig 1 my guess (unless you tell me I am mistaken) is that there are two orientations of the path which should have the same kinetic profile in your model. The one shown, and the other where the order of the four edges on one of the two blue pentamers is clockwise instead of anti-clockwise.

2) When constructing minimum energy paths, the manuscript states that (line 168) for more then one choice of lowest energy configuration, an arbitrary choice is made. However, this choice could impact on the choices further down the path, resulting in lower drops in free energy further down the path which may be kinetically less favorable. It is probably not too much of an issue with the doedec since it is small, has short assembly with limited options, but some discussion about this issue would be nice. Potential fixes would be a breadth first search of the assembly paths, keeping the n lowest energy at each pentamer addition. This is also a simple check to see if there is more then one embedding which has the same energy at each step (point 1 above).

Points 1) and 2) of reviewer 2, which are related, are cogent for which we thank the referee. The limitation in the original manuscript to just one minimum energy assembly pathway was motivated by experimental evidence that viral assembly is dominated by a limited number of pathways (ref: Asor et al.). However, given that the model already represents a very significant simplification, this restriction to a single pathway is indeed a serious flaw and it turned out that it was not necessary. After discussing the comments of the referee, we discovered a computational method that allows inclusion of *all* possible minimum energy assembly pathways. We have adopted this method, redid all computations and rewrote the paper accordingly. The new method gave very interesting results. We encountered a kinetic version of an order-disorder transition. For higher energy activation barriers, there is a selective "phase" where the molecules with large wrapping number and small MLD win packaging contests. For such molecules, assembly is dominated by a small number of minimum energy pathways and the description in the previous manuscript was OK. However, for lower energy activation barriers there is now also an entropy-dominated, non-selective state where molecules with large MLD and small wrapping number and with a very large number of competing minimum energy pathways win the packaging competition for entropic reasons.

We added in the methods section an explanation how the spanning trees were constructed. A Python notebook is available on request.

3) The choice of energy parameters needs more justification. In Zlotnick (1994) the protein-protein interaction energy was chosen such that fully assembled empty particles were obtained at thermodynamic equilibrium with almost no free capsid. The critical threshold value for this was around -2.8 kCal/Mol (which depends on concentration of total protein - your c0). I would have thought that it would be ideal to be somewhere around this value since empty particles for most viruses form readily at appropriate concentration in vitro, but it is not clear where your parameters lie. (see point 4 in MINOR) Some more clarification here would be appreciated.

We agree and added a subsection just before the conclusion that discusses how the energy parameters of the model were estimated from in-vitro assembly experiments. The critical threshold of Zlotnick (1994) for empty capsids corresponds to $E_0 = 4.28$ in units of k_BT while the numerical work we used $E_0 = 4.0$ in units of k_BT.

MINOR

1) The model for RNA interactions with the capsid has a Hamiltonian path on a dodecahedron and

a dodecahedron for the protein shell. In Dykeman et al. the capsid was a dodecahedron and the Hamiltonian path was on an icosahedron. This was due to RNA-CP interactions being modeled as occurring at the centers of the capsids. Here both the protein cage and Hamiltonian path cage are dodehaedron, with RNA interactions on the edges. There is nothing wrong with this per se, but I would clarify that this models the type of situation in Nodaviridae. Some discussion of how the model could be used in viruses such as MS2 would also be useful.

The referee is right. The -- rather hybrid -- model borrows features from the MS2 phage virus and from the Nodaviridae. Moreover, ssRNA viruses of interest generally have T=3 capsids (or even higher T numbers) while in the model the capsid is that of a T=1 virus. There choices were made to minimize the mathematical complexity, though constructing all spanning trees and solving the model numerically still involved extensive computational labor. An advantage of the model is that a Brownian dynamics simulation has been done on a very similar model for the case of a linear genome (Perlmutter et al.). The new text now points out these limitations in the conclusion where we also discuss various improvements of the model that would make it more realistic.

2) A brief discussion on why (mathematically) every spanning tree for the dodecahedron must have 20 nodes and 19 links would be useful.

The number of nodes is twenty because a spanning tree must visit all the vertices, of which there are twenty. The number of links is nineteen because in any connected tree graph the number of links is one less than the number of nodes. This is now pointed out in the text.

3) Similarly, the wrapping number and its connection with the number of pentamer edges occupied by RNA should also be discussed more. There are important nuances between the linear case and the branched case, i.e. in the linear case, pentamers must have either 2/3/4 edges occupied by RNA, for any embedding (as the Hamiltonian path requirement enforces this when there are only 2 end nodes as in the linear case) where as for the branched case, there could be embeddings which have pentamers with 0/1/2/3/4 edges occupied (due to > 2 end nodes). An end node is a vertex on the spanning tree with only one link emanating from it.

We agree and included the remarks of the referee nearly verbatim.

4) The values for the energy parameters (in particular e1,e2,e3,e4) are somewhat convoluted into dependencies on D,c0 etc.. I would suggest also computing the values for e1-e4 for your examples and state concentrations of co in equivalent values of uM, as this will allow for comparisons with expected concentration in cells and in vitro experiments.

They certainly are. We removed the unnecessary parameters e3 and e4 to simplify the discussion. In the subsection mentioned earlier we discuss how the energy parameters can be measured and what the typical solution concentrations are (though in mg/ml).

5) For the model, is there an intermediate step (where RNA-CP contact is formed followed by CP-CP contact) or do both RNA-cp and CP-CP contacts occur in one step?

In the proposed model, as well as in the Dykeman et al scenario and in the original work by Klug on TMV assembly, RNA-CP and CP-CP (or oligomer-oligomer) contacts are established at the same time during viral assembly but in the antenna and en-masse scenarios that is not the case.

6) Some more discussion on the nucleation energies and a comparison between the different spanning trees would be interesting.

We made a comparison between the nucleation energies of spanning trees with different MLD and wrapping numbers. In general, increasing the MLD and reducing the wrapping number increases the nucleation energy but with definite exceptions.

7) There was no discussion (as promised) about ways to incorporate RNA conformation entropy into the model in the conclusions.

This is now discussed in the conclusion.

8) *Some discussion on how RNA secondary structure can be mapped onto your spanning trees (e.g. where are single stranded and double stranded areas on the tree, where are LD interactions and local hairpins). I think your model is currently applicable to Nodaviridae, but I would be interested in a brief discussion of how it could be applied to MS2.*

For the Nodaviridae, double-stranded DNA lines the edges of pentamer, which is consistent with the model, but for just about any other case a different geometrical measure will have to replace the wrapping number concept, as discussed in the conclusion. We are currently investigating this for the MS2 case.
